# Numerical investigation of the effect of substrate surface roughness on the performance of zigzag graphene nanoribbon field effect transistors symmetrically doped with BN

**DOI:** 10.3762/bjnano.5.168

**Published:** 2014-09-17

**Authors:** Majid Sanaeepur, Arash Yazdanpanah Goharrizi, Mohammad Javad Sharifi

**Affiliations:** 1 Department of Electrical and Computer Engineering, Shahid Beheshti University, Velenjak Ave., Tehran, Postal Code: 1983963113, Iran

**Keywords:** boron nitride, non-equilibrium Greens function (NEGF), on-/off-current ratio, substrate roughness, zigzag graphene nanoribbon field effect transistor (ZGNRFET)

## Abstract

The performance of field effect transistors comprised of a zigzag graphene nanoribbon that is symmetrically doped with boron nitride (BN) as a channel material, is numerically studied for the first time. The device merit for digital applications is investigated in terms of the on-, the off- and the on/off-current ratio. Due to the strong effect of the substrate roughness on the performance of graphene devices, three common substrate materials (SiO_2_, BN and mica) are examined. Rough surfaces are generated by means of a Gaussian auto-correlation function. Electronic transport simulations are performed in the framework of tight-binding Hamiltonian and non-equilibrium Green's function (NEGF) formalisms. The results show that with an appropriate selection of the substrate material, the proposed devices can meet the on/off-current ratio required for future digital electronics.

## Introduction

Field effect transistors (FETs) with a 10 nm gate length are stipulated by the International Technology Roadmap for Semiconductors (ITRS) for the year 2020 [1]. Regarding the Si scaling limits, it is obvious that for device lengths to be scaled below 10 nm new materials and device concepts are inevitable. Graphene is one of the most promising materials as a substitute for Si nanotransistors [1–2]. Due to its remarkable electronic properties, graphene has attracted significant attention from physicists and device engineers [3–4]. Unmodified graphene sheets do not have a band gap, therefore graphene transistors are not suitable for digital applications for which a minimum band gap of 360 meV is needed [5–6]. Nevertheless, graphene nanoribbons (GNRs) smaller than 15 nm show a bandgap inversely proportional to the width of the GNR [7–11]. Armchair graphene nanoribbons (AGNRs) are non-magnetic. Zigzag graphene nanoribbons (ZGNRs), however, have a spin-polarized ground state and a high density of states localized at the zigzag edges of the ribbon [8,12–14]. Nonetheless, the energy of the spin-polarized state is only 20 meV per edge atom lower than that of the non-polarized state [15–16]. In addition, the spin-polarized state becomes unstable with respect to the non-polarized state in the presence of a ballistic current through the GNR [17]. Moreover, the magnetization is theoretically proved to be forbidden in one- and two-dimensional systems at finite temperatures [18]. While most transistors work at a finite temperature (typically room temperature), one can consider only the non-polarized metallic state of ZGNRs for the investigation of ZGNR-based transistors [19–20]. The electronic properties of B–C–N nanostructures and their application in electronic devices have been extensively studied [20–24]. It is shown that a gap can be opened in the GNR band structure by doping with BN [25–26]. This band gap is attributed to the broken symmetry of the graphene sub-lattices [25]. BN-doped ZGNRs may be used as a channel material for nanometer-sized conventional field effect transistors. Although the one-atom thick nature of GNRs makes them highly sensitive to their environmental surroundings, for example, the substrate material, phonons from the substrate and charged impurities, here, we only consider the effect of the substrate material. Any substrate material has some surface roughness (SR). Substrate surface roughness induces a conformal surface roughness on the GNR placed on top of it [27]. It has been shown that SR decreases the mobility of both armchair and zigzag GNRs [28]. Also the performance of armchair GNRFETs (AGNRFETs) is significantly degraded by SR [29]. In this work the device performance of symmetrically BN-doped zigzag graphene nanoribbon field effect transistors (s-BN-ZGNRFETs) is numerically studied for the first time. SiO_2_, BN and mica substrates with SR amplitudes of approximately 168–360, 75 and 24 pm, respectively, are considered [27,30–31]. Rough surfaces are generated by means of Gaussian auto-correlation functions [32–33]. The electronic transport calculations are performed in the framework of a non-equilibrium Green's function and tight-binding Hamiltonian. Because of the statistical nature of SR an ensemble average is taken over a large number of devices to obtain averaged device characteristics.

## Model and Methods

The sp^2^ hybridization of carbon atoms in the GNR lattice is preserved in the presence of B or N dopants [34]. Therefore one can model the electronic structure of BN-doped ZGNRs with a first nearest neighbor tight-binding Hamiltonian [34]. The hopping parameters between C, B and N atoms and the on-site energies in place of B and N atoms are set according to [35]. Due to the Gaussian distribution of the experimental data, a two-dimensional Gaussian auto-correlation function is used as a generator of surface roughness [27,30–31]:

[1]
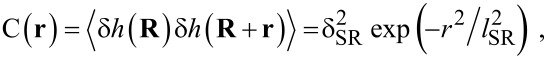


where *h*(**r**) is the variation of the surface height at point **r**. The values δ_SR_ and 
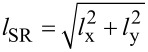
 represent the root mean square and correlation length of the height fluctuations, respectively. The p_z_ orbital of carbon is responsible for the electronic properties of graphene. Although the SR modulates both the distance and angle between the p_z_ orbitals through, stretching and bending of C–C bonds, respectively, the effect of bending is negligible [36]. Therefore, in order to incorporate the effect of the SR into the tight-binding Hamiltonian, the GNR lattice is mapped on the C(**r**) surface and the heights of carbon atoms are modulated accordingly. The lattice mismatch of graphene and BN is 1.7%, which is negligible in comparison to the displacement of atoms due to SR [37]. Therefore, the hopping parameters between displaced atoms of any type can be written as [38–39]:

[2]
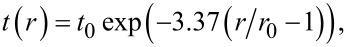


where *t* and *t*_0_ represent the hopping integral between two displaced atoms and the first nearest neighbor hopping parameter of the flat GNR, respectively. All bond lengths of the flat BN-doped ZGNR should be equal to the C–C bond length represented by *r*_0_ = 1.42 Å. Having the system Hamiltonian at hand, the retarded Green's function can be written as [40]:

[3]
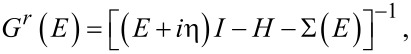


where η is an infinitesimal positive number. The non-Hermitian self-energy matrix, Σ(*E*) = Σ_S_(*E*) + Σ_D_(*E*), represents the escape rates of electrons from the device into the semi-infinite source and drain ideal leads. The self-energy matrices are calculated through a highly convergent recursive method [41]. The transmission as a function of energy is obtained through the Landauer formula [42]:

[4]
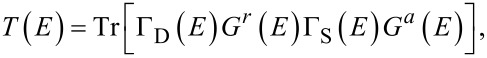


in which *G^a^*(*E*) = [*G^r^*(*E*)]^†^ is the advanced Green's function and Γ*_i_* (*i* = S,D) represents the level broadening due to the coupling of the device to the leads:

[5]
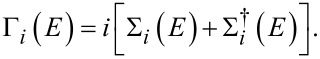


Finally, the current through the device is calculated through the Landauer formula [33–34]:

[6]
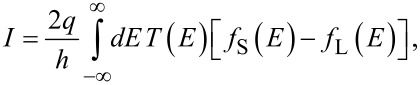


in which *q* is the electron charge and *f* represents the Fermi–Dirac distribution function. To shed more light on the physics behind the device performance, the concept of the transport gap has been employed, which is defined as twice the energy in which the transmission drops below 10^−2^ [43–44]. A more detailed description of the methods is available in [28]. The transistor is considered to be in the on-state when the voltage difference between the gate and the source contacts (*V*_GS_) equals the maximum supplied voltage to the device (*V*_DS_), which is considered to be 1 V herein. The off-state is defined as the device state at *V*_GS_ = 0. The on- and off-current of the device are defined as the current flow from the source to the drain in the on- and off-state, respectively [45].

## Results and Discussion

**Figure 1:**
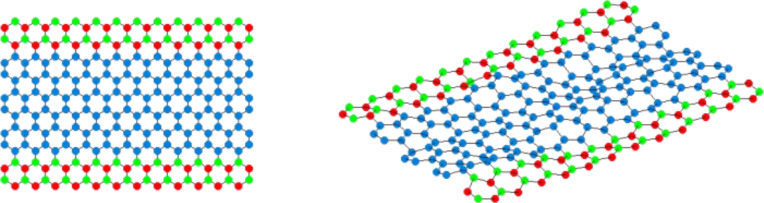
Top view of a 5h-2BN-ZGNR (left panel) and the same GNR with surface roughness (right panel). The blue circles represent carbon atoms while boron and nitrogen dopants are shown in green and red, respectively.

A typical sample of s-BN-ZGNR with a width of five hexagons of atoms (5h) is shown in Figure 1 with (right) and without (left) surface roughness. As depicted, the one hexagonal chain of atoms at each edge along the transport direction is doped with BN dimers, namely, a 5h-2BN-ZGNR structure.

**Figure 2:**
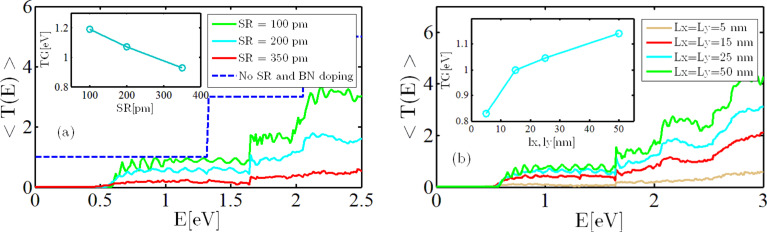
(a) Averaged transmission of 10 nm length 4h-2BN-ZGNR as function of the energy for various SR amplitudes (*l*_x_ = *l*_y_ = 25 nm). The dashed-blue line belongs to the flat channel (a 4h-2BN-ZGNR without SR). The inset shows the transport gap versus SR amplitude. (b) Averaged transmission probability of 10 nm length 4h-2BN-ZGNR as function of the energy for various SR correlation lengths (δ_SR_ = 200 pm). The inset shows the transport gap versus SR correlation length.

Figure 2a illustrates the average transmission of a 10 nm length 4h-2BN-ZGNR versus energy for various SR amplitudes. As shown by increasing the roughness amplitude, the transmission decreases due to an increased SR scattering rate [28]. The inset of Figure 2a shows that by increasing the roughness amplitude from 100 to 350 pm, the BN-doping induced transport gap decreases from 1.19 to 0.99 eV due to decreased average hopping between edge atoms of ZGNR and BN dopants. The average transmission of a 10 nm length 4h-2BN-ZGNR with 200 pm SR amplitude for various correlation lengths is shown in Figure 2b. As depicted by decreasing the correlation length, the transmission decreases due to the larger scattering rate of the rougher surface [28]. The transport gap however decreases by decreasing the correlation length (see inset of Figure 2b). The roughness-dependent behavior of the transport gap in ZGNRs (insets of Figure 2a and Figure 2b) is in contrast to the undoped AGNRs for which the transport gap remains constant when the roughness parameters are modulated [28].

**Figure 3:**
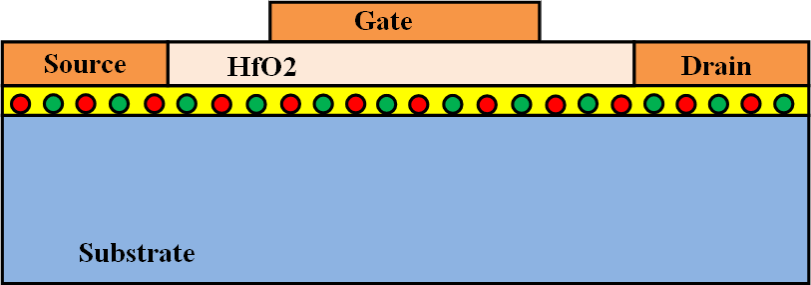
Schematic representation of the simulated device structure. The gate insulator is assumed to be 2.5 nm thick HfO_2_ (κ = 25). Source and drain contacts are heavily doped extensions of the channel without SR.

**Figure 4:**
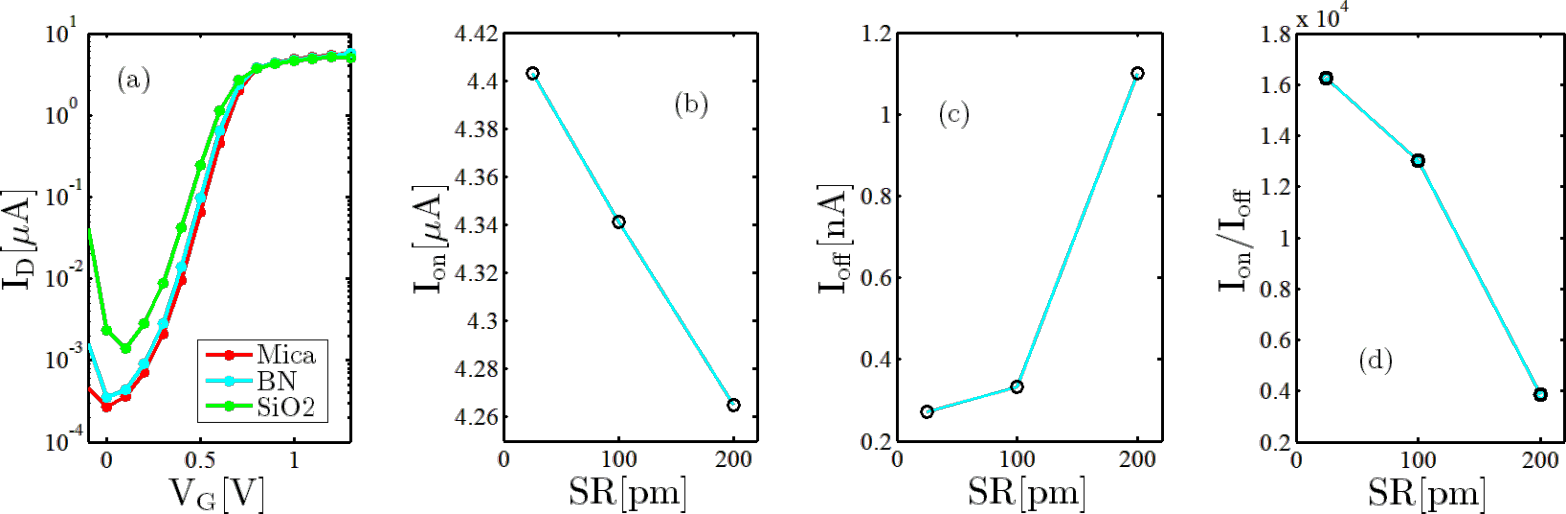
(a) Averaged transfer characteristic (b) on-current (c) off-current and (d) on-/off-current ratio of a 1.56 × 10 nm^2^ 4h-2BN-ZGNRFET for various substrate materials. In panels (b) to (d) the horizontal axis shows the SR amplitude. The correlation length is considered to be 25 nm for SiO_2_ and 5 nm for mica and BN [27,30–31].

The structure of the simulated device is depicted in Figure 3. Figure 4a shows the average transfer characteristics of a 1.5 × 10 nm^2^ 4h-2BN-ZGNRFET for various substrate materials in logarithmic scale. As shown by increasing the SR amplitude from 25 pm (mica) to 200 pm (average SR amplitude of a SiO_2_ substrate) the on-current decreases from 4.4 to 4.2 μA while the off-current increases from 0.27 to 1.1 nA (see Figure 4b and Figure 4c). The observed decrease in the on-current is due to larger SR scattering rate on rougher surfaces while the off-current increases due to the SR-induced decrease in the transport gap, both stated above. Moving from mica to SiO_2_, both the roughness amplitude and correlation length increase. The former tends to roughen the surface while the latter tends to flatten it. Therefore one can conclude from Figure 4b–d that for the substrate materials selected for this work the SR amplitude effect dominates that of the correlation length. Figure 4d shows that the on/off-current ratio for a 10 nm long 4h-2BN-ZGNRFET on a SiO_2_ substrate does not meet the required amount of 10^4^ for digital transistors [5]. It is possible however to improve the device performance in terms of on/off-current ratio by increasing the width of the BN-doped regions.

**Figure 5:**
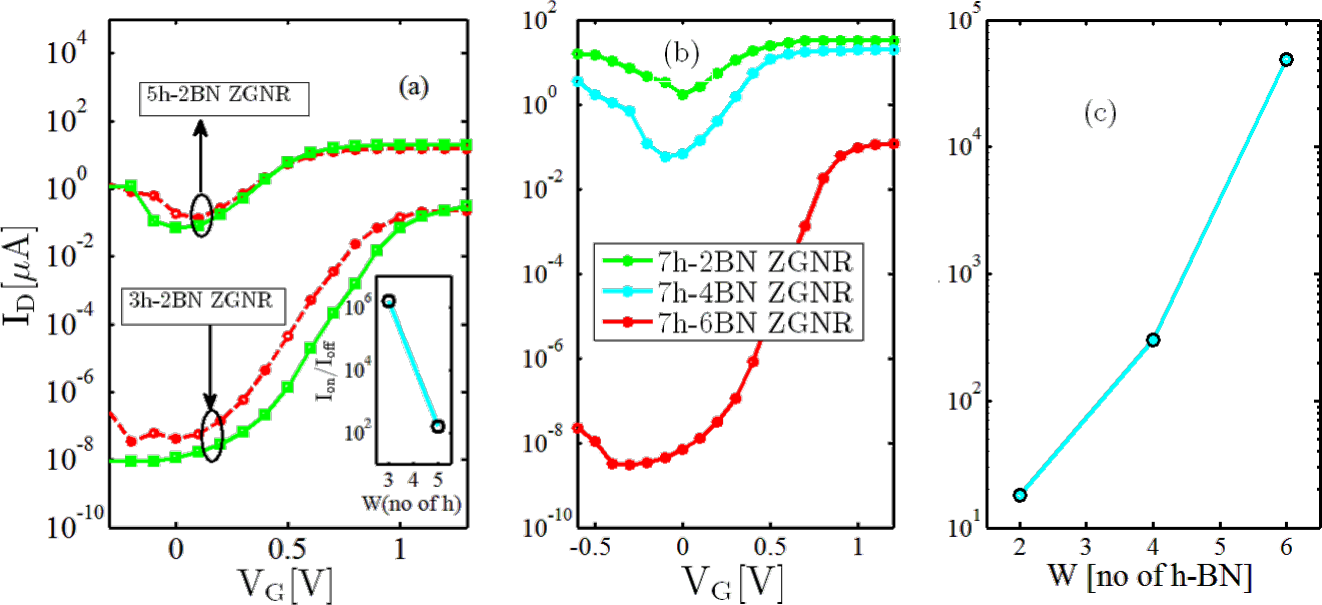
(a) The average transfer characteristic of 10 nm length devices with SiO_2_ substrate for different device widths but the same BN widths (3h-2BN and 5h-2BN) with (red) and without (green) SR. The inset shows the on-/off-current ratio of rough devices with respect to device width. (b) The average transfer characteristic and (c) the on-/off-current ratio of a 2.84 × 10 nm^2^ device (7h) with SiO_2_ substrate for various BN widths.

Figure 5a shows the average transfer characteristics of two 10 nm length devices with different device widths but the same BN widths (3h-2BN and 5h-2BN) with (red) and without (green) SR. As shown, by increasing the width from 1.14 to 2 nm, both the on- and the off-current increase. The on-current increases due to an increased number of conducting channels while the observed increase in the off-current is due to decreased HOMO–LUMO gap (and, consequently, a decreased transport gap). As a result of this, the on/off-current ratio decreases from 1.6 × 10^6^ in the 3h-2BN device to 156 in the 5h-2BN device (see inset of Figure 5a). To resolve the problem of an increased off-current in wider devices, the width of the BN-doped region must be increased. Figure 5b shows the average transfer characteristic of a 2.84 × 10 nm^2^ device (7h) with a SiO_2_ substrate for various BN widths. By doping the three hexagonal chain from each edge of the ZGNR (7h-6BN-ZGNRFET) the on/off-current ratio reaches 4.86 ×10^4^, which falls within the acceptable range for digital transistors (see Figure 5c).

## Conclusion

In summary, symmetrical BN doping was applied on ZGNRs to open a necessary gap for use in digital electronic applications. Next, the device performance of FETs with symmetrically BN-doped ZGNRs as a channel material was numerically studied. Effect of the surface roughness for several common substrate materials on the device performance was explored. To achieve the minimum on/off-current ratio of 10^4^ for s-BN-ZGNRFETs, an appropriate substrate material must be chosen. In general it is possible to widen the device and the BN-doped portion of the channel to achieve larger a on/off-current ratio. Results show that by symmetrical doping ZGNRs with BN and appropriate selection of substrate material in terms of the surface roughness, ZGNRFETs can meet the required on/off-current ratio for future digital electronics.
